# *Phellinus linteus* activates different pathways to induce apoptosis in prostate cancer cells

**DOI:** 10.1038/sj.bjc.6603595

**Published:** 2007-01-30

**Authors:** T Zhu, J Guo, L Collins, J Kelly, Z J Xiao, S-H Kim, C-Y Chen

**Affiliations:** 1 Department of Radiation Oncology, Beth Israel Deaconess Medical Center, Harvard Medical School, Boston, MA, USA; 2 Department of Biochemistry, Boston University School of Medicine, Boston, MA USA; 3 Laboratory of Angiogenesis and Chemoprevention, Graduate School of East-West Medical Science, Kyunghee University, Boston, South Korea

**Keywords:** PL, apoptosis, caspases, ER

## Abstract

It is known that polysaccharides extracted from the *Phellinus linteus* (PL) mushroom possess antitumour activity. We previously have demonstrated that high doses of PL render murine or human lung cancer cells susceptible to apoptosis. However, the molecular mechanisms of PL-mediated apoptosis have not been fully explored. In this study, we demonstrate that LNCaP cells expressing the androgen receptor (AR) are highly susceptible to apoptosis in response to treatment with high doses of PL. In this process, caspase 8 and its downstream effectors (such as BID), as well as ER stress-related, apoptotic signalling, are activated. In contrast, a moderate amount of apoptosis occurs in PC3 cells (that lack AR) after the same treatment, which does not activate ER-mediated apoptotic signalling. We also show that, in the process of PL-induced apoptosis, caspase 2 is induced in LNCaP cells, but not in PC3 cells. However, LNCaP cells that express a mutated AR or LNCaP cells treated with a caspase 2 inhibitor blocked ER stress-induced apoptotic signals. The magnitudes of the induction of apoptosis in these cells are comparable with what occurred in the PC3 cells. The data demonstrate that high doses of PL activate the AR-dependent and independent apoptotic pathways. Our study also suggests that caspase 2 is a key target in the determination of the susceptibility of prostate cancer cells to PL-induced apoptosis.

*Phellinus linteus* (PL) is among a number of well-known medicinal mushrooms from Asian countries, which have been taken orally since ancient times as a health-promoting dietary supplement and an adjuvant to combat viral and bacterial infections. PL, after purification, shows a relatively homogeneous molecular weight distribution on gel permeation HPLC and is estimated to be around 150 kDa from the retention time on HPLC pullulan molecular markers (Song *et al*, 1995). The main components of PL are polysaccharides (Song *et al*, 1995; Lorenzen and Anke, 1998; Borchers *et al*, 1999; Han *et al*, 1999). Many studies demonstrated that polysaccharides from various substances, including PL, are remarkably effective in inhibiting the growth of tumours without toxic side effects. Studies also showed that polysaccharides in PL are able to suppress tumours, either indirectly by enhancing the host's immune system or directly by inducing apoptosis in tumour cells (Chihara *et al*, 1969; Chung *et al*, 1982; Cun *et al*, 1994; Wasser, 2002; Collins *et al*, 2006; Guo *et al*, 2006). Therefore, the antitumour, antiangiogenic and immunomodulatory effects of PL are potential areas for developing novel pharmaceutical products. However, the underlying molecular mechanisms of the antitumour effects of PL have not yet been fully explored.

Changes in the androgen receptor (AR) have been indicated to contribute to the development of prostate cancer and are a serious challenge to effective treatment. Mutations in the AR can increase its affinity for ligand binding, permitting activation by non-androgenic hormones or even antagonists (Nelson *et al*, 2003; Debes and Tindall, 2004). Overexpression of AR increases the sensitivity of prostate cancer cells to low levels of androgens, which promotes androgen-independent growth (Linja *et al*, 2001; Chen *et al*, 2004; Debes and Tindall, 2004). Studies also showed that AR, as a crucial factor, determines the molecular alterations required for the development of refractory prostate cancer (Debes and Tindall, 2004). In general, the AR is bound to heat shock proteins, in the cytoplasm. Upon binding to the active androgen dihydrotestosterone (DHT), the receptor dissociates from the heat shock proteins, and translocates to the nucleus (Feldman and Feldman, 2001; Grossmann *et al*, 2001; Chen *et al*, 2004). In the nucleus, AR dimerises and binds to androgen-response elements to initiate the transcription of genes required for growth. With the progression of the degree of the malignancy, prostate cancer loses expression of AR and abrogates the hormone dependency. These observations indicate that a major challenge in the treatment of prostate cancer, especially the hormonally refractory form, is to understand the genetic and cellular alterations associated with the susceptibility of prostate cancer to apoptosis. We have demonstrated that PL, at low doses, can synergize with doxorubicin (an anticancer drug) to induce apoptosis in LNCaP cells (Collins *et al*, 2006). However, it is not clear how PL transmits apoptotic signals in these cells.

Apoptosis can be triggered by a variety of internal or external signals. Caspases, a highly conserved family of cysteine proteases, are key apoptotic effectors and play a critical role in apoptosis through a cascade of cleavage events (Schmitz *et al*, 2000; Shi, 2002). Caspases exist as dormant proenzymes in cells and are activated through proteolysis. Upon apoptotic stimulation, cleaved caspase 8, as an initiator, leads to the activation of immediate downstream effector caspases 3 and 7, resulting in BID cleavage and cytochrome *c* release (Thornberry *et al*, 1997; Bhornberry and Lazebnik, 1998). The release of cytochrome c from the mitochondria to the cytosol causes the formation of the apoptosome, followed by activation of caspase 9, which, in turn, results in a caspase chain reaction (Susin *et al*, 1999). Among caspase family members, caspase 2 is unique because it has features of both the long prodomain of upstream caspases and the optimal recognition motif of downstream caspases (Harvey *et al*, 1997; Droin *et al*, 2001). The prodomain is essential for the dimerisation and autoprocessing of precursor caspase 2, which allow caspase 2 to be directly activated and further initiate the caspase cascade in response to various apoptotic stimuli (Harvey *et al*, 1997; Droin *et al*, 2001).

The endoplasmic reticulum (ER) is the main intracellular organelle in charge of proper folding and maturation of transmembrane and secretory proteins (Kaufman 1999; Patil and Walter, 2001; Demaurex and Distelhorst, 2003). Studies have demonstrated that the ER functions as a sensor of cellular stress to maintain homeostasis in cells (Ma and Hendershot, 2001, 2004; Rutkowski and Kaufman, 2004; Schroder and Kaufman, 2005). De-regulation of ER functions is often associated with diseases ranging from diabetes to neurodegenerative illnesses (Forman *et al*, 2003; Takuma *et al*, 2005). ER stress can cause changes in intracellular calcium levels, nutrient deprivation altered glycosylation or lipid overload (Ma and Hendershot, 2001, 2004; Rutkowski and Kaufman, 2004; Schroder and Kaufman, 2005). To compensate for ER stress, the unfolded protein response is activated in cells, during which the expression of certain proteins resident in the ER (such as BiP or ATF-4) is elevated (Ma and Hendershot, 2004; Sriburi *et al*, 2004). Persistent protein overload in the ER can trigger apoptosis (Herr and Debatin, 2001; Breckenridge *et al*, 2003). Damage to the ER activates certain caspase family members, including caspase 2 (Morishima *et al*, 2002; Chae *et al*, 2004).

Previous studies, including ours, demonstrated that PL possesses antitumour properties (Chihara *et al*, 1969; Chung *et al*, 1982; Cun *et al*, 1994; Wasser 2002; Collins *et al*, 2006; Guo *et al*, 2006). We recently reported that PL, at low doses, can sensitise prostate cancer cells for cytotoxicity induced by anticancer drugs (such as doxorubicin) (Collins *et al*, 2006). We also showed that high doses of PL can elicit apoptosis in mouse and human lung cancer cells (Guo *et al*, 2006). In the present study, we further investigated the molecular mechanisms of PL-mediated apoptosis in prostate cancer cells. LNCaP cells express the AR; PC3 cells do not. We demonstrated that high doses of PL could induce large numbers of LNCaP cells to undergo apoptosis, whereas the magnitude of apoptosis in PC3 cells was moderate. During the process of PL-induced apoptosis, caspase 8 and its downstream effectors (such as caspase 3 and BID) were cleaved and further activated in LNCaP and PC3 cells. However, the expression of caspase 2 was elevated in LNCaP cells only, but not in PC3 cells. ER stress-related apoptotic signals were also activated in LNCaP cells. ER-mediated apoptosis was blocked by the addition of caspase 2 inhibitor to LNCaP cells as well as abolished in a strain of LNCaP cells expressing a mutated AR. Our study implies that high doses of PL are able to elicit multiple apoptotic signalling pathways in prostate cancer cells, and that caspase 2, acting downstream of the AR, sensitises prostate cancer cells to PL-induced apoptosis.

## MATERIALS AND METHODS

### Cells and reagents

*Phellinus linteus* powder was purchased from Panbio-Tech (Taejon, South Korea) and purified using ethanol precipitation methods followed by DEAE-cellulose and gel permeation chromatography (Song *et al*, 1995). The purified components of PL consist mostly of polysaccharides. The media for cell culture including DMEM, antibiotics (penicillin and streptomycin) and trypsin-EDTA were purchased from Invitrogen (Carlsbad, CA, USA). Antibodies were purchased from Pharmingen (Palo Alto, CA, USA). Human prostate cancer cell lines LNCaP and PC3 were purchased from American Type Culture Collection (Manassas, VA, USA) and were cultured in Dulbecco's modified Eagle's medium supplemented with 10% heat-inactivated fetal calf serum, 2 mM L-glutamine, 100 U ml^−1^ of penicillin and 100 g ml^−1^ of streptomycin. Normal human prostate epithelial PrEC cells (Cambrex, NJ, USA) were cultured in the PrEGM medium (Cambrex, NJ, USA). All the antibodies used were purchased from BD Biosciences (San Diego, CA, USA). The caspase 2 inhibitor was purchased from Calbiochemical (San Diego, CA, USA).

### Luciferase assay

Cells were cotransfected with 15 *μ*g of the luciferase construct and 2 *μ*g of *β*-gal (an internal control). Forty-eight hours later, the cells were grown in Charcol medium for 6 h and stimulated with DHT. Subsequently, the luciferase activity was analysed.

### DNA fragmentation analysis

A flow cytometric analysis was performed using a FACScan (Becton Dickenson, Mountain View, CA, USA). The data analysis was performed using the Cell-Fit software program (Becton Dickenson). Cell-Fit receives data from the flow cytometer and provides real-time statistical analysis, computed at 1 s intervals, and also discriminates doublets or adjacent particles. Cells with subG_0_–G_1_ DNA contents after staining with propidium iodide were counted as apoptotic cells. In brief, 48 h following the treatment, the cells were harvested by trypsinisation, washed with 1 × cold PBS and then fixed in 70% cold ethanol. Afterwards, the cells were stained with 0.1 mg ml^−1^ propidium iodide containing 1.5 mg ml^−1^ RNase. Following incubation at room temperature for 2 h, the DNA contents of the cells were tested by a Becton Dickinson FACScan machine (BD Biosciences) and evaluated with BD FACStation software CellQuest.

### Annexin V assay

Following treatments, cells (1 × 10^6^) were washed twice with cold PBS and stained with Annexin V-FITC using the Annexin kit (BD Biosciences) to detect apoptotic cells using a flowcytometer.

### Immunoblot analysis

After lysing cells in lysis buffer (150 mM NaCl, 0.1% Nonidel P-40, 0.1% SDS, 50 mM Tris, 50 *μ*g ml^−1^ phenylmethylsulfphonyl fluoride, 10 *μ*g ml^−1^ aprotinin, 5 *μ*g ml^−1^ leupeptin, 0.1 *μ*g ml^−1^ NaF), whole-cell lysates containing equal amounts of total proteins were prepared and separated on an SDS–PAGE gel. Subsequently, proteins were blotted to a nitrocellulose membrane. After blocking the membrane in the blocking solution (5% non-fat milk in 1 × TBS-T (10 mM Tris-HCl, pH 8.0, 150 mM NaCl and 0.05% Tween 20)) for 1 h at room temperature, immunoblotting was performed. The proteins of interest were detected by autoradiography after treating the membrane with Western lightning Western blot chemiluminescence reagent (Perkin Elmer Life Sciences, Boston MA, USA). The membranes were stripped and re-probed with a *β*-actin antibody to ensure equal loading of proteins. Each immunoblot analysis was repeated more than once to ensure reproducibility.

### Caspase activity analysis

Caspase 8 and 2 colorimetric assay kits (Bio Vision) were used to measure the activities of caspase 8 and 2 in cell lysates. The assays are based on spectrophotometric detection of the chromophore *p*-nitroanilide (*p*NA) after cleavage from the labelled substrate IETD-*p*NA for caspase 8 or VDVAD-*p*NA for caspase 2. The *p*NA light emission is quantified using a spectrophotometer. Briefly, cells (0.5 × 10^6^), with or without treatment with PL, were lysed in 1% Triton X-100 buffer (pH 7.2) containing protease inhibitors and subsequently subjected to the assay.

### Soft agar assay

Petri dishes were first layered with 0.6% basal agar dissolved in DMEM medium containing 10% fetal calf serum. Cells were mixed in 0.33% agar dissolved in DMEM medium containing PL (1 mg ml^−1^). The plates were incubated at 37°C in a humidified atmosphere for 14 days. Fresh medium (2 ml) containing PL was added to the cultures every 3 days.

### Statistics

Means and standard deviations of the results of the experiments were computed. Standard deviations are displayed as error bars in the figures.

## RESULTS

### Different susceptibilities of LNCaP and PC3 Cells to apoptosis induced by PL mushroom

**Figure 1:**
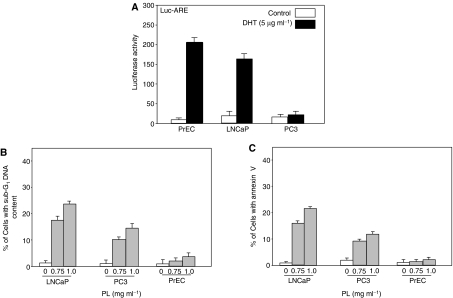
AR function and PL-mediated DNA fragmentation. (**A**) PrEC, LNCaP and PC3 cells were cotransiently transfected with an AR-responsive element-luciferase and *β*-gal (internal control) constructs. Forty-eight hours post-transfection, cells were cultured in the medium without growth factors for 6 h and stimulated with DHT for 24 h. Subsequently, luciferase activity was analysed. Error bars represent the standard deviation over five independent experiments. (**B**) The cells were treated with different doses of PL for 48 h, and the percentage of the cells with fragmented DNA was analysed by a flow cytometer. Error bars represent the standard deviation over five independent experiments. (**C**) After treatment with PL, the percentage of the cells stained with Annexin V was measured.

It is known that PL possesses antitumour properties, which have been linked to its polysaccharide content (Song *et al*, 1995). We recently demonstrated that PL, at low doses (<0.5 mg ml^−1^), synergises with doxorubicin (an anticancer drug) to induce apoptosis in prostate cancer LNCaP cells (Collins *et al*, 2006). To explore the molecular mechanisms of PL-induced apoptosis and the effect of the AR on this process, human prostate epithelial PrEC cells and prostate cancer LNCaP and PC3 cells were used. The functionality of AR in the cells was first confirmed using a luciferase assay. A construct containing the AR responsive element fused with luciferase was transiently transfected into PrEC, LNCaP and PC3 cells, and the luciferase activity was assayed after DHT treatment (Figure 1A). AR on the surface of PrEC and LNCaP cells, but not PC3 cells, was responsive to DHT. The ability of PL at high doses (0.75 and 1 mg ml^−1^) to induce apoptosis was then tested (Figure 1B). Forty-eight hours after PL treatment, a DNA fragmentation assay was conducted. A few untreated PrEC, LNCaP or PC3 cells had fragmented DNA. A large number of LNCaP cells and a lesser amount of PC3 cells underwent PL-induced apoptosis in a dose-dependent fashion. However, very few PrEC cells underwent apoptosis in response to the treatment, which is consistent with our previous observation that PL has little toxicity on normal cells (Song *et al*, 1995; Guo *et al*, 2006). To further confirm the occurrence of apoptosis induced by high doses of PL, an Annexin V assay was conducted (Figure 1C), and similar results were obtained. Overall, the results suggest that AR might be a factor in the regulation of the susceptibility of prostate cancer cells to PL-induced apoptosis.

Next, we performed a soft agar assay to determine whether PrEC cells resemble normal, non-transformed cells, and if high doses of PL are able to kill prostate cancer cells and prevent them from forming colonies in soft agar (Table 1). PrEC cells did not form colonies in soft agar. In comparison, untreated LNCaP or PC3 cells formed colonies in soft agar medium. The numbers of colonies generated from PC3 cells were higher than those from LNCaP cells, indicating that PC3 cells are more malignant. However, the addition of PL dramatically reduced the ability of LNCaP cells to form colonies in a dose-dependent manner. The same doses of PL only moderately prevented PC3 cells from forming colonies. The results further suggest that PL, at high doses, induces apoptosis and blocks prostate cancer growth.

### Caspases 8/3 and BID are activated in LNCaP and PC3 cells in response to high-dose PL treatment

**Figure 2:**
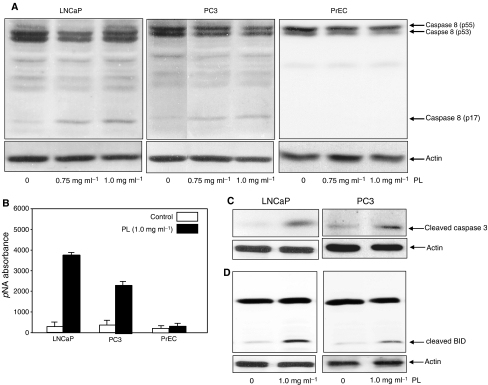
Activation of caspase 8, caspase 3 and BID in response to treatment with PL. (**A**) After treating with different concentrations of PL, cell lysates from PrEC, LNCaP and PC3 cells were prepared. Expression of the activated form of caspase 8 was determined by Western blot. Equal loading of total proteins was verified by *β*-actin expression. (**B**) After PL treatment, cell lysates were prepared to analyse caspase 8 activity (**B**–**D**). Following the treatments, lysates were prepared to analyse the presence of the cleaved, active forms of caspase 3 or BID. Equal loading of total proteins was verified by *β*-actin expression.

Caspase family members are important factors in the induction of apoptosis triggered by various apoptotic stimuli (Schmitz *et al*, 2000; Shi, 2002). The activation of caspases involves a series of protein cleavages resulting in small, active fragments for initiating apoptosis (Thornberry *et al*, 1997; Bhornberry and Lazebnik, 1998). Caspase 8 has been shown to be one of the initiators of the caspase cascade (Thornberry *et al*, 1997; Bhornberry and Lazebnik, 1998). We tested whether caspase 8 is activated by high doses of PL in LNCaP and PC3 cells. Immunoblot analysis was conducted to detect the existence of the cleaved, active p17 fragment of caspase 8 (Figure 2A). The active fragment of caspase 8 was not revealed by anti-caspase 8 Ab in PrEC cells with or without PL treatment. Caspase 8 also could not be detected in untreated LNCaP and PC3 cells. In contrast, the active form of the protease was present in treated LNCaP cells, and present in lesser amounts in PC3 cells. To further confirm the activation of caspase 8 induced by PL, the enzymatic assay of caspse 8 was analysed (Figure 2B). The results were consistent, in that caspase 8 activity, after PL treatment, was higher in LNCaP cells than that in PC3 cells, and was undetectable in PrEC cells.

Caspase 3 and BID are the downstream effectors in the caspase 8-mediated cascade (Thornberry *et al*, 1997; Bhornberry and Lazebnik, 1998). The status of these caspase family members, following treatment with high doses of PL, was examined by immunoblot analysis (Figure 2C and D). The active form of caspase 3 and the cleaved fragment of BID were not seen in untreated LNCaP or PC3 cells, but were revealed by the corresponding antibodies in both cell lines treated with 1 mg ml^−1^ of PL. The results indicate that caspase 8 and its downstream effectors participate in PL-induced apoptosis in prostate cancer cells, whether they express AR or not.

### Caspase 2 is upregulated in LNCaP but not in PC3 Cells after treatment with high doses of PL

**Figure 3:**
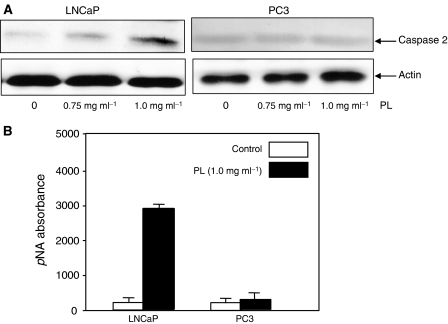
Caspase 2 activation in response to different doses of PL treatment in prostate cancer cells. (**A**) After treating LNCaP and PC3 cells, cell lysates were prepared and subsequently analysed for the expression of caspase 2. Equal loading of total proteins was verified by *β*-actin expression. (**B**) After PL treatment, cell lysates were prepared to analyse caspase 2 activity.

From the analysis of its primary structure, caspase 2 contains a long NH_2_-terminal prodomain, which is used by apoptotic stimuli to initiate apoptosis, and an optimal recognition motif of downstream caspases to execute apoptotic processes (Harvey *et al*, 1997; Droin *et al*, 2001). To further search for the cause of the different sensitivities of LNCaP and PC3 cells to PL-induced apoptosis, the expression of caspase 2 was examined by immunoblotting (Figure 3A). A baseline expression of caspase 2 was detected by anticaspase 2 Ab in untreated LNCaP cells, and the levels of the protease were significantly increased after treatment with high doses of PL in a dose-dependent manner. However, the baseline expression of caspase 2 in PC3 cells did not change after PL treatment. The activity of caspase 2 after PL treatment was also tested by the enzymatic assay (Figure 3B). The activity of caspase 2 was dramatically induced by PL in LNCaP cells but not in PC3 cells.

**Figure 4:**
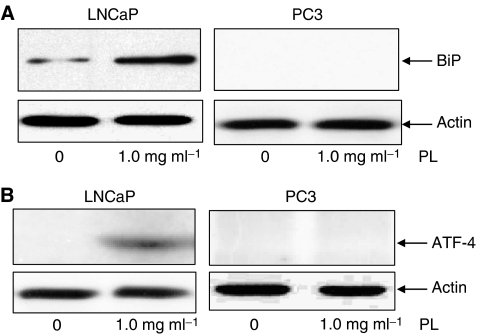
Expression of the ER proteins BiP and ATF-4 in PL-treated prostate cancer cells. LNCaP and PC3 cells were untreated or treated with 1 mg ml^−1^ of PL for 24 h and lysates were prepared. Subsequently, immunoblotting was conducted to detect BiP (**A**) and ATF-4 (**B**) expression. Equal loading of total proteins was verified by *β*-actin expression.

The unfolded protein response occurs in ER stress-induced apoptosis and is reflected by increases in ER proteins (Patil and Walter, 2001). ER stress-induced apoptosis has been demonstrated to be regulated in part by caspases (Morishima *et al*, 2002; Chae *et al*, 2004). As caspase 2 is differentially expressed in LNCaP and PC3 cells, we tried to determine whether caspase 2, through the ER, is sensing PL treatment. We first tested the expression of ER proteins, such as BiP and ATF-4, by immunoblotting (Figure 4). A baseline level of BiP expression was detected in untreated LNCaP cells, and this ER protein was induced by PL (Figure 4A). In comparison, PL had no effect on BiP expression in PC3 cells. The same expression patterns of ATF-4 were obtained from LNCaP and PC3 cells (Figure 4B). The data suggest that high doses of PL elicited the unfolded protein response in the cells expressing AR, but not in those without a functional AR.

### Effect of AR or caspase 2 on PL-induced apoptosis

**Figure 5:**
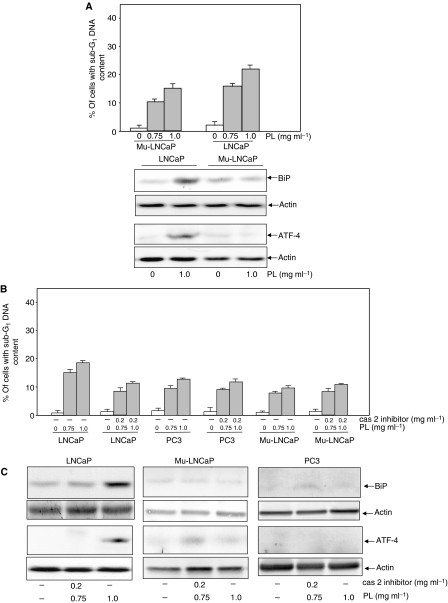
Effect of mutant AR or caspase 2 inhibitor on PL-induced apoptosis. (**A**) After treating LNCaP or LNCaP cells expressing a mutated AR with different doses of PL, the percentage of DNA fragmentation was measured by a flow cytometer (upper panel). The lysates from untreated or treated cells were also prepared, and immunoblotting was conducted to detect the expression of BiP and ATF-4 (lower panel). Equal loading of total proteins was verified by *β*-actin expression. (**B**) LNCaP, mutant LNCaP and PC3 cells were treated with a caspase 2 inhibitor before PL treatment. Subsequently, the percentage of DNA fragmentation was measured by a flow cytometer. (**C**) The lysates from untreated or treated LNCaP or mutant LNCaP cells were also prepared for immunoblotting to detect the expression of BiP and ATF-4. Equal loading of total proteins was verified by *β*-actin expression.

It has been reported that the AR, by repressing the *cis*-element of the caspase 2 promoter, regulates caspase 2 expression (Rokhlin *et al*, 2005). Our results presented above indicate the involvement of AR or caspase 2 in PL-induced apoptosis in LNCaP cells. To test the linear relationship between these two molecules under such apoptotic conditions, an LNCaP cell line that possesses a non-functional AR was employed. After treatment of LNCaP and its mutant cells with 0.75 or 1 mg ml^−1^ of PL, a DNA fragmentation assay was conducted (Figure 5A, upper panel). LNCaP cells underwent apoptosis after PL treatment in a dose-dependent fashion. The magnitude of apoptosis induced by PL was notably lower in the LNCaP mutants than in the wild-type cells. An Annexin V assay was also performed, and similar results were obtained (data not shown). Furthermore, the expressions of BiP and ATF-4 were examined by immunoblot analysis (Figure 5A, lower panel). The ER proteins were induced by PL in LNCaP cells but not in the mutant cells. The data indicate that AR is an important regulator in apoptosis and is induced by high doses of PL.

Next, we tested the effects of a caspase 2 inhibitor on PL-induced apoptosis. LNCaP, mutant LNCaP and PC3 cells were treated with a caspase 2 inhibitor before the high-dose PL treatment. Subsequently, the percentage of DNA fragmentation was measured (Figure 5B). The magnitude of PL-induced apoptosis in LNCaP cells was partially suppressed in the presence of the caspase 2 inhibitor, which is similar to what occurs in PL-treated PC3 cells. In comparison, the inhibitor had a minimal effect on apoptosis induced by PL in PC3 or mutant LNCaP cells. An Annexin V assay was also performed and similar results were obtained (data not shown). The expressions of BiP and ATF-4 were tested with or without the addition of a caspase inhibitor in LNCaP or mutant LNCaP cells by immunoblotting (Figure 5C). The inhibitor blocked the expressions of these ER-stress proteins in LNCaP cells. Overall, the data suggest the existence of a linear relationship between AR and caspase 2 in response to PL treatment, which sensitises ER-regulated apoptotic signalling.

## DISCUSSION

The major findings in our current study are that high doses of PL elicit two major apoptotic pathways in prostate cancer cells: a caspase 8-induced cascade and the unfolded protein response. The activation of caspase 8 and its downstream effectors may be a general effect of PL on tumours. The unfolded protein response in the ER appears to be prostate cancer specific, and is regulated by the AR and caspase 2. Our study suggests a molecular target of PL, which provides a better understanding of the potential of PL to treat prostate cancer.

It is known that PL is not toxic in general (Song *et al*, 1995). We have demonstrated that PL, at high doses, causes lung epithelial cells to arrest in the G_1_ phase of the cell cycle by blocking the expression of cyclin D_1_ and its further interaction with cell cycle-dependent kinases 4 and 6 (Guo *et al*, 2006). It is possible that treatment with PL elicits G_1_ checkpoint control in normal prostate epithelial cells, such as PrEC, resulting in the cessation of cell cycle progress. In tumour cells, cell cycle checkpoints are often impaired. Under such conditions, high doses of PL become apoptotic and activate the cell death programme in prostate cancer cells.

It has been reported that caspase 2 expression is regulated by the AR. As an unusual member of the caspase family, caspase 2 possesses features that can both initiate and execute programmes of cell death (Harvey *et al*, 1997; Droin *et al*, 2001). Activation of caspase 2 results in a complex that consists of the death-domain-containing protein and the adapter protein. It has also been shown that activated caspase 2 can directly cause the cleavage of BID (Harvey *et al*, 1997; Droin *et al*, 2001). In addition, studies have demonstrated that caspase 2, upon different apoptotic stimulations, re-distributes to the nuclei or other subcellular membrane compartments to participate in the execution of the cell death programme (Mancini *et al*, 2000; Chae *et al*, 2004; Rokhlin *et al*, 2005). Our results here demonstrate the interconnection between AR and caspase 2 in the regulation of PL-induced apoptosis in prostate cancer cells. Studies to identify molecular targets in the cooperation between AR and caspase 2 during PL-induced apoptosis are under way.

In the progression of prostate cancer, AR is often mutated or its expression is lost, which plays an important role in the development of the resistance of cancer cells to treatment. Therefore, the discovery of molecular targets to sensitise apoptotic signalling pathways has important therapeutic implications. Genetic targeting or pharmacological manipulation of caspase family members and their regulators/modulators would provide better clinical strategies. It has been shown that overexpression of caspase 7 in LNCaP cells could effectively induce apoptosis, and this has been very promising clinically (Marcelli *et al*, 1999). Our present study indicates that caspase 2 is an intracellular target for upregulating the susceptibility of PC3 cells or prostate cancer cells containing a mutated AR to PL-induced apoptosis.

Activation of caspase family members is at the core of apoptosis, representing a point of intersection of various apoptotic pathways. Tumour necrosis factor or Fas/CD95 receptors, mitochondrial proteins (such as cytochrome *c*) and granzyme are able to induce cell death through activation of the caspase cascade (Schmitz *et al*, 2000; Shi, 2002). Damage to or stress in the ER or Golgi has been shown to be able to trigger apoptosis (Herr and Debatin, 2001; Breckenridge *et al*, 2003). Studies have shown that the unfolded protein response or lack of calcium is responsible for ER-mediated apoptosis (Breckenridge *et al*, 2003). ER stress has been demonstrated to cause the translocation of certain caspases to the ER or Golgi and the execution of apoptosis there. Calcium released from the ER during times of ATP deficiency is an important element in apoptosis induced by ischaemia–reperfusion injury. It is possible that PL treatment upregulates and activates caspase 2 that subsequently translocates to the ER and causes the unfolded protein response, resulting in apoptosis.

Caspases 8, 6 and 3 are major players in caspase-induced apoptosis (Nicholson and Thomberry, 1997). We demonstrated that PL, at low doses, is able to synergise with anticancer drugs (such as doxorubicin) for the induction of apoptosis in LNCaP cells (Collins *et al*, 2006). We also demonstrated that high doses of PL elicit a caspase cascade in human and murine lung cancer cells, but not in normal lung epithelial cells, in which caspase 3, caspase 8 and BID are activated (Guo *et al*, 2006). In this study, we further demonstrate that treatment with high doses of PL can activate caspase 8-initiated apoptotic signalling in both AR-expressing (LNCaP) and AR-null (PC3) prostate cancer cells. It seems that the high doses of PL, by activating the caspase 8-regulated signalling pathway, are generally toxic to various types of tumours and have no effect on normal cells.

The successful treatment of prostate cancer requires identification of specific intracellular targets for sensitising the tumour to apoptosis. Our previous study demonstrated that PL, at low doses, acts as an enhancer to sensitise anticancer drug-mediated, apoptotic signalling, and this sensitization can be obtained at subtoxic concentrations of the drug. In this study, we showed that high doses of PL can mobilize multiple apoptotic signalling pathways to cause different degrees of cytotoxic effects on prostate cancer cells, depending on the expression of AR. We also conclude that caspase 2, in an AR-dependent fashion, may be a specific intracellular switch for the regulation of the susceptibility of tumour cells. Our data suggest that PL, by modulating caspase activity, can be developed for more efficient therapies against refractory prostate cancer.

## Figures and Tables

**Table 1:** Colony formation of PrEC, LNCaP and PC cells in soft agar in the presence of PL

**Cell types**	**Treatment**	**No of colonies**	**s.d.**
PrEC	No treatment	0	±0
LNCaP	No treatment	154	±7.5
LNCaP	PL (0.75 mg ml^−1^)	17	±4
LNCaP	PL (1.0 mg ml^−1^)	5	±2
PC3	No Treatment	208	±1.8
PC3	PL (0.75 mg ml^−1^)	106	±8
PC3	PL (1.0 mg ml^−1^)	68	±5

The cells were cultured in soft agar in the absence or presence of different concentrations of PL for 2 weeks. Every 3 days, fresh growth medium containing PL was added into the cultures. s.d.: standard deviation over five independent experiments.
